# Oral Dysaesthetic and Perceptual Disorder, A Distinct Subset of Chronic Orofacial Pain Without Burning Symptoms: A Case–Control Study

**DOI:** 10.1111/joor.13945

**Published:** 2025-01-27

**Authors:** Gennaro Musella, Federica Canfora, Vito Carlo Alberto Caponio, Emmanouil Vardas, Maria Kouri, Nikolaos Nikitakis, Giuseppe Troiano, Massimo Aria, Luca D'Aniello, Lorenzo Lo Muzio, Michele Davide Mignogna, Daniela Adamo

**Affiliations:** ^1^ Department of Clinical and Experimental Medicine University of Foggia Foggia Italy; ^2^ Department of Neuroscience, Reproductive Sciences, and Dentistry University of Naples Federico II Naples Italy; ^3^ Department of Oral Medicine and Pathology and Hospital Dentistry National and Kapodistrian University of Athens Athens Greece; ^4^ Department of Economics and Statistics University of Naples Federico II Naples Italy; ^5^ Department of Social Sciences University of Naples Federico II Naples Italy; ^6^ Department of Life Science, Health, and Health Professions Link Campus University Rome Italy

**Keywords:** burning mouth syndrome, dysgeusia, misperception, somatic symptom disorder, xerostomia

## Abstract

**Background:**

According to the ICOP 2020, Burning Mouth Syndrome (BMS) is a chronic orofacial pain disorder characterised by an intraoral burning sensation, which represents the main diagnostic criterion. However, some patients experience other symptoms such as xerostomia, taste alterations, and globus, without the burning sensation (non‐BMS).

**Objective:**

This study aims to explore non‐BMS as a distinct subclinical entity by comparing the classical BMS with this new group of patients in a case–control study, addressing gaps in current diagnostic criteria.

**Methods:**

Eighty‐three non‐BMS patients were compared with an equal number of BMS patients matched for age and sex. Data on sociodemographic and risk factors, systemic comorbidities and drug intake, symptom patterns, psychological profiles were collected and statistically analysed.

**Results:**

No significant differences between BMS and non‐BMS groups were found in sociodemographic characteristics, comorbidities, drug consumption and extraoral symptoms. Both groups reported similar symptoms patterns, with discomfort intensifying in the evening. Although oral symptoms prevalence varied, with the burning sensation exclusive to BMS patients, none of these differences reached statistical significance. Psychological assessments revealed mild depression (17 [14–19] vs. 17 [14–20], *p*  0.981), mild to moderate anxiety (18 [15–21] vs. 17 [15–20.5], *p* = 0.767), and sleep disturbance (5 [4–7] vs. 8 [5–10], *p* < 0.001) in both groups.

**Conclusion:**

Non‐BMS patients exhibit similarities to BMS patients yet remain unclassified under current diagnostic criteria. Therefore, we have suggested the term “Oral Dysaesthetic and Perceptual Disorder (ODPD)” to define these patients (non‐BMS). This study emphasises the need to expand diagnostic criteria to better identify and manage ODPD patients.

## Graphical abstract

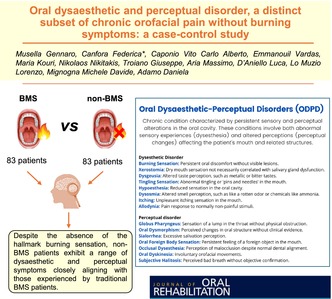

## 1 Background

Burning Mouth Syndrome (BMS) is a chronic, complex, and idiopathic orofacial pain disorder affecting the oral cavity, with an overall prevalence ranging from 1.73% to 7.72% in the general population and clinical settings, respectively [1]. This condition predominantly occurs in postmenopausal females, with a male‐to‐female ratio varying from 3:1 to 16:1, and it is most commonly observed in individuals aged between the fifth and seventh decades of life [2]. Historically, BMS has been referred to by numerous other names based on the location or quality of pain, such as stomatodynia, stomatopyrosis, scalded mouth syndrome, burning mouth condition, sore mouth, glossodynia, glossopyrosis, sore tongue and sore mouth [3].

According to the International Classification of Orofacial Pain (ICOP 2020) [4], BMS is defined as intraoral burning pain or dysaesthesia recurring daily for more than 2 h per day and for more than 3 months, without any identifiable causative lesions and in the absence of local or systemic pathological changes. The ICOP 2020 highlights that burning pain is a defining feature of BMS, often accompanied by other somatosensory symptoms such as subjective xerostomia, dysesthesia and altered taste. Additionally, psychosocial comorbidities commonly observed in other chronic pain conditions are also frequently present in BMS, underscoring its complex clinical presentation. However, the symptomatology of BMS is intricate and varies, with burning sensation being the most reported symptom, often supplemented by a broad range of other oral extraoral dysaesthetic or perceptual disturbances alongside burning or pain [5]. Among these, common oral symptoms include subjective salivary alterations such as xerostomia and sialorrhea, taste disturbances, an intraoral foreign body sensation, globus, and itching [6]. Taste alterations, such as dysgeusia, distorted perceptions like a metallic or bitter taste, and phantom sensations, are often underestimated but can adversely impact food intake and choices, potentially resulting in malnutrition and immune dysfunction [7]. Moreover, BMS patients might report a globus sensation, described as the feeling of a lump or foreign body in the throat which can be intermittent or constant, contributing to anxiety and frequent throat clearing. Further symptoms can include intraoral foreign body sensation, oral dysmorphisms such as a change in tongue or gums morphology or colour, occlusal dysaesthesia, persistent burdensome or dull ache, allodynia, hypoesthesia, oral dyskinesia, and subjective halitosis [5, 8, 9]. The additional symptoms frequently complicate the disease and contribute to the diagnostic delay of BMS because many instrumental exams and evaluations by other specialists are required to exclude possible related local and systemic conditions with the same symptoms [6]. Therefore, BMS may remain under‐recognised and under‐appreciated by both dental and medical professionals for a long time [10]. It took an average of 30 months from the onset of symptoms until a definitive diagnosis of BMS was achieved and every patient consulted almost three specialists, receiving several misdiagnoses before the proper diagnosis. This diagnostic delay common for BMS becomes further prolonged when a patient reports only additional symptoms. Indeed, when a patient reports burning pain along with one or more additional symptoms, it is possible to include them in the BMS diagnosis [6]. Conversely, if a patient does not report burning but exhibits one symptom, such as globus or occlusal dysaesthesia, or xerostomia a specific diagnosis of globus or occlusal dysaesthesia or xerostomia can be made. However, in our clinical practice, many patients have reported multiple oral symptoms but without burning pain, and with similar clinical and laboratory findings with the classic BMS patients. When multiple symptoms are present simultaneously such as dysgeusia, globus and subjective change in tongue morphology or other combinations like xerostomia, sialorrhea and tingling, a clear diagnosis cannot be made according to the ICOP 2020 and International Classification of Headache Disorders, 3rd edition (ICHD‐3) criteria [4]. These classifications specify that a BMS diagnosis requires the presence of a daily recurring intraoral burning sensation or dysaesthesia, with oral pain needing to be of burning quality and felt superficially in the oral mucosa. It is important to first rule out a range of intraoral conditions that can mimic or contribute to oral burning sensations, such as candidiasis, xerostomia, mucosal diseases (e.g. oral lichen planus), allergies to dental materials, mechanical irritation from dental appliances, nutritional deficiencies, and hormonal changes [11]. Only after these potential causes are excluded can a diagnosis of BMS be considered, underscoring the complexity and careful evaluation required in distinguishing BMS from other oral conditions.

This study aimed to address a gap in current diagnostic criteria by characterising a group of patients with oral sensory symptoms and dysperceptions who lack the burning sensations typical of BMS (non‐BMS), and by comparing them to classic BMS patients. Although these non‐BMS patients do not experience burning pain, many present with a range of symptoms that significantly impact their quality of life and complicate both diagnosis and management. To date, no studies have systematically characterised this subgroup or examined the clinical distinctions between BMS and non‐BMS patients. The lack of recognition of non‐BMS as a distinct diagnostic entity contributes to diagnostic delays and suboptimal care.

The primary objective of this study is to characterise and compare the sociodemographic characteristics, comorbidities, medication use, frequency and patterns of oral symptoms, psychological profiles, and sleep disturbances in BMS and non‐BMS patients, determining whether there are clinically significant differences that justify recognising non‐BMS as a distinct condition.

## 2 Methods

### 2.1 Study Design

An observational, cross‐sectional, case–control study was conducted at the Oral Medicine Department of the University of Naples “Federico II” over a period from May 2021 to December 2023. The study received approval by the Ethical Committee of the University (Approval Number: 251/19—date of approval February 20, 2019), ensuring that it adhered to ethical principles of the World Medical Association Declaration of Helsinki. Furthermore, the methods employed in this study followed the Strengthening of the Reporting of Observational Studies in Epidemiology (STROBE) guidelines for observational studies.

### 2.2 Patients Enrolment

All potentially eligible participants were invited to participate in the study, and written informed consent was obtained from all subjects involved in the study. The patient selection process involved two steps. First, a random sample of 100 non‐BMS patients and 400 BMS patients was drawn. To ensure comparability between the groups, we applied statistical matching using the nearest neighbour distance method [12, 13, 14]. This method was selected to minimise confounding effects from key sociodemographic factors. Through this approach, we identified two subgroups of 83 individuals each, matched precisely for age and sex. This careful matching process ensured comparable groups, thereby enhancing the reliability of our analysis.

### 2.3 Inclusion and Exclusion Criteria

The BMS group inclusion criteria followed the ICOP 2020, 1st edition: [4]

- Patients experiencing the symptoms of oral burning recurring daily for > 2 h per day for > 3 months without any clinical mucosal alterations,
- Patients with normal blood test findings (including blood count, blood glucose levels and glycated haemoglobin, serum iron, ferritin, and transferrin).

The inclusion criteria of non‐BMS groups were as follows:

- Patients experiencing oral dysaesthesia without any clinical mucosal alterations,
- Patients without a history of BMS,
- Patients with normal blood test findings (including blood count, blood glucose levels and glycated haemoglobin, serum iron, ferritin and transferrin).

The exclusion criteria for both groups were as follows:

- Patients suffering from diseases that could be recognised as a causative factor of BMS or non‐BMS [15],
- Patients unable to understand the questionnaires,
- Patients having a history of a psychiatric disorder (depression, anxiety disorders, bipolar disorder, schizophrenia, and obsessive‐compulsive disorder) or an organic brain disorder,
- Patients undergoing treatment with systemic psychotropic drugs able to influence mood, perception, cognition and behaviour (antidepressants, antipsychotics, anxiolytics and mood stabilisers),
- Patients with a history of gastroesophageal reflux disease without stable proton pump inhibitor therapy for at least 3 years without recent reflux episodes,
- Patients with a possible correlation between their symptoms and the introduction of a new medication were excluded, as medication‐related side effects are expected to manifest soon after treatment initiation or following adjustments in dosage or type,
- Patients having a history of alcohol or substance abuse, and
- Patients in treatment with systemic drugs possibly associated with oral symptoms, and patients suffering from Obstructive Sleep Apnea Syndrome (OSAS).

## 3 Clinical Evaluation

### 3.1 General Information

An oral medicine specialist (DA) conducted a comprehensive intraoral and extraoral examination for each patient to systematically identify and rule out any oral diseases or conditions that could be associated with oral burning or contribute to altered sensory perception in the oral mucosa. This examination aimed to exclude any causative factors, such as infections, mucosal lesions, dental issues or systemic conditions that might impact oral sensation. By carefully assessing each patient, we ensured that any underlying conditions or potential confounders affecting oral perception were identified and excluded. Data collection was performed through in‐person questionnaires that covered various aspects in terms of dichotomous or multiple variables. Sociodemographic information included age (in years), years of education, marital status (single, married, divorced, widowed), and employment status (employed, unemployed, retired); the number of medical specialists consulted for oral symptoms before our examination, BMI (body mass index, divided into ranges: BMI < 18.5; BMI: 18.5–24.9; BMI: 25.0–29.9; BMI: 30–34; BMI: 35–39.99; BMI > 40), physical activity (yes/no), menopause for women (yes/no), and risk factors including smoking (smokers/never smokers, and frequency: < 5 cigarettes/day; 5–10 cigarettes/day; 10–15 cigarettes/day; > 15 cigarettes/day) and alcohol intake (drinkers/non drinkers, and frequency using a cut‐off of 14 units/week) were also recorded. Additionally, information on systemic comorbidities, Age‐Adjusted Charlson Comorbidity Index (AACCI) [16], and medication usage were collected. The AACCI is a validated scoring system used to predict the 10‐year mortality for a patient who may have a range of comorbidities. Each condition is assigned a score based on its potential to impact patient survival, with age adjustments incorporated to account for increasing risk with advancing age. This index is widely utilised in clinical research and practice to assess the burden of comorbidity and to adjust for confounding in studies of clinical outcomes.

Furthermore, following a comprehensive examination of the patient's medical records related to previous consultations with physicians who specialise in the particular field of the symptoms (neurologists, otolaryngologists, gastroenterologists, gynaecologists, dermatologists, internal medicine specialists and rheumatologists), the extraoral physical symptoms were documented and classified as Medically Unexplained Physical Symptoms (MUEPS) [17], and are as follows: fibromyalgia, tinnitus, irritable bowel syndrome, skin itching, and vulvodynia.

### 3.2 Oral Symptoms

During the medical examination, the type and pattern of oral dysaesthesia was also investigated (whether they remained the same throughout the day, worsened in the afternoon or evening, were worse in the morning, or were continuous or intermittent), any relief experienced during eating, and the most severe symptom reported by each patient. The symptoms have been spontaneously reported by the patients rather than prompting specific questions, to minimise potential suggestion effects. A comprehensive neurological and neuroradiological evaluation was conducted, including MRI imaging of the brain and maxillofacial regions, to rule out conditions such as Parkinson's disease, cerebral palsy, amyotrophic lateral sclerosis, tumours, or other relevant disorders related to the reported symptoms [18, 19]. Additionally, further instrumental tests were performed to confirm the clinical absence of the dysperceptive symptom (Supplementary file—“Symptoms assessment”): dysgeusia was assessed through taste strips and electrogustometry tests [20]; hyposalivation and sialorrhea were evaluated by quantifying salivary flow rates, following standardised procedures based on previously validated research [21]. Additional examinations were also performed, including ultrasound imaging of the major salivary glands using high‐frequency (7–15 MHz) probes to assess structural integrity and help rule out conditions like acute inflammation or sialolithiasis [22]. Allodynia was evaluated by measuring pain intensity on the NRS after stimulation with a cotton swab. A score of three or higher was considered indicative of allodynia, as it reflects pain in response to a normally non‐painful stimulus. Hypoesthesia was evaluated through a mechanical pain threshold test [23]. In patients presenting with pharyngeal globus, a comprehensive evaluation was conducted by both a gastroenterologist and an ear‐nose‐throat specialists. The latter included an examination with flexible fiberoptic nasolaryngoscope with colour video camera to assess for any anomalies [24]. Additionally, a cervical ultrasound was performed to diagnose diseases of the thyroid, salivary glands, lymph nodes and tumours in the head and neck region [25]. Subjective halitosis was assessed by excluding objective causes through a comprehensive evaluation of oral health and hygiene [26]. In this study, we assessed discomfort levels in both BMS and non‐BMS groups using standard pain scales. Since burning sensations in BMS are frequently described as discomfort, these scales are suitable for capturing the overall discomfort experienced by patients across both groups, allowing for a consistent comparison of symptom severity.

### 3.3 Psychological Assessment

This study employed a comprehensive battery of standardised tests to evaluate pain severity, anxiety and depression levels, and sleep quality among the participants [8].

The intensity of pain was measured using the Numeric Rating Scale (NRS), a well‐validated unidimensional scale in which the patient rates the pain from 0 (no oral symptoms) to 10 (worst imaginable discomfort). Instead, the quality of pain was assessed using the short form of the McGill Pain Questionnaire (SF‐MPQ), a multidimensional pain survey, which assesses the sensory, affective and evaluative aspects of the perceived pain. Both questionnaires were administered with a focus on assessing the nature of the discomfort experienced.

The Hamilton Depression Rating Scale (HAM‐D), the most widely used clinician‐administered depression assessment tool, was used to evaluate depressive symptoms, while the Hamilton Anxiety Rating Scale (HAM‐A) was employed to measure both somatic and psychic anxiety. For the HAM‐D, a score above 7 indicates impairment, with scores between 7 and 17 suggesting mild depression, 18–24 pointing to moderate depression, and scores exceeding 24 indicating severe depression. In the HAM‐A evaluation, each item is rated from 0 to 4, and a total score below 17 suggests mild severity, 18–24 indicates mild to moderate severity, and 25–30 points to moderate to severe severity.

The “Pittsburgh Sleep Quality Index” (PSQI) and the “Epworth Sleepiness Scale” (ESS) were employed. The PSQI, a self‐report survey, evaluates subjective sleep quality over the past month. It encompasses seven categories with a total of 19 questions. Each category is scored from 0 (no difficulty) to 3 (severe difficulty). The combined scores of all categories generate a global sleep quality score ranging from 0 to 21, with a score exceeding 5 indicating a potential sleep disturbances with a sensitivity of 90%–99% and a specificity of 84%–87%. The ESS, a self‐administered assessment, assesses an individual's overall level of daytime sleepiness through eight questions rated on a 4‐point Likert scale (0–3). The ESS scores range from 0 to 24, with scores over 10 indicating elevated daytime sleepiness. Additionally, patients were asked how long the reported sleep disturbance had been present, and this was assessed in months.

The CGI Severity of Illness (CGI‐S) scale was used to establish disease severity in both groups.

### 3.4 Sample Size

The sample size was estimated at 169 patients, with 83 in each group, to achieve a statistical power of at least 99% (1‐Beta) and a significance level of no more than 1%. The effect size of 0.77, measured in a previously published research study regarding the SF‐MPQ [27], was used for this calculation. The GPower software (v. 3.1.9) [28] was utilised to determine the sample size.

### 3.5 Data Analysis

The statistical analysis was employed using R software (version 4.4.0). Descriptive statistics, including means, standard deviations (SD), medians, and interquartile ranges (IQR), were calculated to analyse the socio‐demographic data, risk factors, systemic diseases, drugs consumption, age‐adjusted charlson comorbidity index, symptoms, and clinical characteristics between BMS and non‐BMS. Normal distribution was preliminarily assessed performing the Shapiro–Wilk test. Differences in percentages were evaluated using the Pearson Chi‐Square test, and Fisher's exact test was used when the frequency was less than five. The non‐parametric Mann–Whitney *U* test was employed to compare the median scores of clinical parameters (NRS, SF‐MPQ, CGI, HAM‐D, HAM‐A) and sleep parameters (PSQI, ESS). Bonferroni correction was applied to perform multiple statistical tests across different outcome measures [29, 30, 31], and the *p*‐values considered statistically significant were used accordingly.

## 4 Results

A total of 166 BMS patients, 83 BMS and 83 non‐BMS, matched for age and sex, were recruited including 59% males and 41% females for both groups.

Table 1 presents the sociodemographic characteristics, BMI, menopause state, physical activity and habits of the study sample. The average age of patients was comparable between the two groups (BMS: 65.4 ± 15 years; non‐BMS: 65.3 ± 15.2 years; *p* = 0.967). Educational attainment, measured in years, also showed no significant difference. Regarding marital status, a higher percentage of non‐BMS patients were married than BMS patients (80.7% vs. 68.7%; *p* = 0.146), and there were more widowed individuals in the BMS group (15.7% compared to 7.2%), though these differences were not statistically significant. The BMI of patients did not significantly differ between the groups, with both groups predominantly being within the 25.0–29.9 range. Menopausal status and physical activity levels were similarly distributed across both groups, with no significant differences with 14.5% engaging in physical activity and 85.5% not engaging. Risk factors such as smoking and alcohol use were also similar, with slight variations in the percentage of smokers and drinkers but no significant differences overall.

**TABLE 1:** Sociodemographic characteristics, body mass index, menopause state, physical activity, and habits of samples of patients.

Demographic variables	BMS	Non‐BMS	*p*
**Gender**	**Frequency (%)**	**Frequency (%)**	
Male	49 (59.0)	49 (59.0)	1.000
Female	34 (41.0)	34 (41.0)	
**Age (in years)**	**Mean ± SD**	**Mean ± SD**	
	65.4 ± 15	65.3 ± 15.2	0.967
**Education (in years)**	**Mean ± SD**	**Mean ± SD**	
	9.29 ± 4.19	9.51 ± 4.55	0.736
**Family situation**	**Frequency (%)**	**Frequency (%)**	
Married	57 (68.7)	67 (80.7)	0.146
Widowed	13 (15.7)	6 (7.2)	
Single	7 (8.4)	8 (9.6)	
Divorced	6 (7.2)	2 (2.4)	
**Employment**	**Frequency (%)**	**Frequency (%)**	
Retired	32 (38.6)	31 (37.3)	0.299
Employed	31 (37.3)	39 (47.0)	
Unemployed	20 (24.1)	13 (15.7)	
**Body Mass Index (BMI)**	**Mean ± SD**	**Mean ± SD**	
Total	27.1 ± 4.54	26.9 ± 4.07	0.794
	**Frequency (%)**	**Frequency (%)**	
BMI < 18.5	0 (0.0)	1 (1.2)	
BMI: 18.5–24.9	30 (36.1)	27 (32.5)	
BMI: 25.0–29.9	39 (47.0)	41 (49.4)	
BMI: 30–34	8 (9.6)	9 (10.8)	
BMI: 35–39.99	3 (3.6)	3 (3.6)	
BMI > 40	3 (3.6)	2 (2.4)	
**Menopause**	**Frequency (%)**	**Frequency (%)**	
Yes	37 (44.6)	37 (44.6)	1.000
No	46 (55.4)	46 (55.4)	
**Physical activity**	**Frequency (%)**	**Frequency (%)**	
Yes	12 (14.5)	12 (14.5)	1.000
No	71 (85.5)	71 (85.5)	
**Risk factors**			
**Smoking**	**Frequency (%)**	**Frequency (%)**	
Smokers	25 (30.1)	22 (26.5)	0.731
Never smokers	58 (69.9)	61 (73.5)	
Very light smokers (< 5 cigarettes)	6 (24.0)	2 (9.1)	0.161
Light smokers (5–10 cigarettes)	5 (20.0)	3 (13.6)	
Moderate smokers (10–15 cigarettes)	10 (40.0)	7 (31.8)	
Heavy smokers (> 15 cigarettes)	4 (16.0)	10 (45.5)	
**Alcohol use**	**Frequency (%)**	**Frequency (%)**	
Drinkers	15 (18.1)	13 (15.7)	0.836
Not drinkers	68 (81.9)	70 (84.3)	
Light/moderate drinkers (≤ 14 units/week)	9 (60.0)	10 (76.9)	0.435
Heavy drinkers (> 14 units/week)	6 (40.0)	3 (23.1)	

*Note:* The significant difference between means was measured by the Student *t*‐test.   The significant difference between percentages was measured by the Pearson chi‐square test. Significant *p* ≤ 0.05.

Abbreviations: BMI, Body Mass Index; BMS, burning mouth syndrome; non‐BMS, non burning mouth syndrome.

Table 2 illustrates the prevalence of systemic diseases, AACCI, and patient drug consumption. No statistically significant differences in the prevalence of systemic diseases were found between the two groups. However, hypercholesterolemia and gastroesophageal reflux disease were more prevalent in the BMS group compared to the non‐BMS group (hypercholesterolemia: 54.2% vs. 36.1%, *p* = 0.029; gastroesophageal reflux disease: 28.9% vs. 12%, *p* = 0.012). The AACCI scores were similar between both groups (BMS: 2 [1–3]; non‐BMS: 2 [0–3]; *p* = 0.885) indicating similar comorbidity burdens. Regarding drug consumption, the use of angiotensin receptor blockers was higher in the BMS group (27.7% vs. 13.3%, *p* = 0.033). Other medications, including statins, antiplatelets, proton pump inhibitors, beta‐blockers, and calcium channel blockers, did not show relevant differences in usage between the groups.

**TABLE 2:** The prevalence of systemic diseases, Age‐Adjusted Charlson Comorbidity Index and the drugs consumption of samples of patients.

	BMS	Non‐BMS	*p*
**Systemic diseases**	**Frequency (%)**	**Frequency (%)**	
Essential Hypertension	48 (57.8)	35 (42.2)	0.062
Hypercholesterolemia	45 (54.2)	30 (36.1)	0.029
Gastroesophageal reflux disease	24 (28.9)	10 (12)	0.012
Solid tumour Localised	11 (13.3)	4 (4.8)	0.102
Benign prostatic hypertrophy	7 (8.4)	11 (13.3)	0.455
Congestive heart failure	6 (7.2)	5 (6)	1.000
Liver Disease	6 (7.2)	2 (2.4)	0.277
Hypothyroidism	5 (6)	9 (10.8)	0.403
Myocardial infarction	5 (6)	5 (6)	1.000
Chronic obstructive pulmonary disease	4 (4.8)	4 (4.8)	1.000
Psoriatic Arthritis	3 (3.6)	1 (1.2)	0.620
Connective tissue disease	1 (1.2)	0 (0)	1.000
Atrial fibrillation	1 (1.2)	1 (1.2)	1.000
Hyperthyroidism	1 (1.2)	1 (1.2)	1.000
Hypoacusis	1 (1.2)	1 (1.2)	1.000
Osteoporosis	1 (1.2)	0 (0)	1.000
Peripheral vascular disease	0 (0)	1 (1.2)	1.000
Cerebrovascular accident or TIA	0 (0)	1 (1.2)	1.000
Chronic kidney disease	0 (0)	1 (1.2)	1.000
Endocrine disease	0 (0)	2 (2.4)	0.497
**Age‐Adjusted Charlson Comorbidity Index**	**Median [IQR]**	**Median [IQR]**	
AACCI	2 [1–3]	2 [0–3]	0.885
**Drug consumption**	**Frequency (%)**	**Frequency (%)**	
Statins	30 (36.1)	23 (27.7)	0.318
Antiplatelets	24 (28.9)	19 (22.9)	0.479
Angiotensin receptor blockers	23 (27.7)	11 (13.3)	0.033
Proton pump inhibitors	21 (25.3)	18 (21.7)	0.715
Beta blockers	18 (21.7)	10 (12)	0.146
Calcium Channel blockers	10 (12)	6 (7.2)	0.431
Diuretics	10 (12)	8 (9.6)	0.804
Ezetimibe	7 (8.4)	2 (2.4)	0.168
ACE‐inhibitors	6 (7.2)	9 (10.8)	0.590
Levothyroxine	4 (4.8)	11 (13.3)	0.102
Blood thinner	3 (3.6)	3 (3.6)	1.000
Bisphosphonate	0 (0)	1 (1.2)	1.000

*Note:* Systemic diseases, significant with Bonferroni correction *p* ≤ 0.003; Age‐Adjusted Charlson Comorbidity Index, significant *p* ≤ 0.05; Drug consumption,significant with Bonferroni correction *p* ≤ 0.004. Abbreviations: ACCI, Age‐Adjusted Charlson Comorbidity Index; BMS, burning mouth syndrome; non‐BMS, non burning mouth syndrome.

Table 3 presents the analysis of pain, depression, anxiety, sleep disturbance, CGI‐S, prevalence of MUEPS, the median duration of the disease onset, the number and type of consultations. The SF‐MPQ was significantly higher in the BMS group (12 [9.5–18] vs. 9 [7–12], *p* < 0.001) suggesting that burning may contribute to worsening of perception of illness. Conversely, sleep quality, measured by the PSQI, was better in the BMS group (8 [6–10] vs. 9 [8–11], *p* = 0.012) and the ESS score was significantly lower in the BMS group, indicating less daytime sleepiness (5 [4–7] vs. 8 [5–10], *p* < 0.001). Notably, sleep disturbance preceded oral symptoms by approximately 5 years in both groups. MUEPS were significantly more prevalent in the BMS group (28.9% vs. 14.5%, *p* = 0.037), with fibromyalgia being notably higher (9.6% vs. 1.2%, *p* = 0.034). Referral patterns to various specialists, including dentists, physicians, maxillofacial surgeons, and others, showed no significant differences, indicating similar healthcare‐seeking behaviours among patients with BMS and non‐BMS.

**TABLE 3:** Analysis of pain, depression, anxiety, sleep disturbance, clinical global impression, medically unexplained extraoral physical symptoms, disease onset, and number and type of consultations of the samples of patients.

	BMS	Non‐BMS	
**Clinical parameter**	**Median [IQR]**	**Median [IQR]**	***p***
**Pain/** **Discomfort**			
NRS	10 [9–10]	10 [8–10]	0.944
SF‐MPQ	12 [9.5–18]	9 [7–12]	< 0.001^a^
**Psychological assessment**			
HAM‐D	17 [14–19]	17 [14–20]	0.981
HAM‐A	18 [15–21]	17 [15–20.5]	0.767
**Sleep**			
PSQI	8 [6–10]	9 [8–11]	0.012
ESS	5 [4–7]	8 [5–10]	**< 0.001** ^a^
Sleep disturbance onset preceding symptoms (years)	5[3–10]	5[3–7]	0.504
**CGI‐S**	5 [4–5]	5 [4–5]	0.146
**MUEPS**	**Frequency (%)**	**Frequency (%)**	***p***
Total	24 (28.9)	12 (14.5)	**0.037**
Irritable bowel syndrome	15 (18.1)	7 (8.4)	0.108
Fibromyalgia	8 (9.6)	1 (1.2)	0.034
Tinnitus	6 (7.2)	3 (3.6)	0.496
Vulvodynia	2 (2.4)	0 (0)	0.497
Skin itching	1 (1.2)	2 (2.4)	1.000
	**Median [IQR]**	**Median [IQR]**	***p***
**Disease onset (months)**	12 [11–36]	12 [12–24]	0.536
**Referrals**	**Frequency (%)**	**Frequency (%)**	
Dentist	76 (91.6)	74 (89.2)	0.793
Physician	58 (69.9)	50 (60.2)	0.254
Maxillofacial surgeon	20 (24.1)	18 (21.7)	0.854
Gastroenterologist	13 (15.7)	14 (16.9)	1.000
Otolaryngologist	10 (12)	19 (22.9)	0.101
Neurologist	5 (6)	7 (8.4)	0.766
Psychiatrist	4 (4.8)	6 (7.2)	0.746
Dermatologist	2 (2.4)	7 (8.4)	0.168

*Note:* A significant difference between the percentages was measured by Fisher's exact test. IQR is the interquartile range. The significant difference between medians was measured by the Mann–Whitney *U* test.

Abbreviations: BMS, burning mouth syndrome; CGI‐S, clinical global impression severity of illness; ESS, Epworth Sleepiness Scale; HAM‐A, Hamilton Rating Scale for Anxiety; HAM‐D, Hamilton Rating Scale for Depression; MUEPS, medically unexplained extraoral physical symptoms; non‐BMS, non‐burning mouth syndrome; NRS, Numeric Rating Scale; PSQI, Pittsburgh Sleep Quality Index; SF‐MPQ, Short form of Mcgill Pain Questionnaire.

^a^ Pain significant with Bonferroni correction 0.01. Sleep significant with Bonferroni correction 0.03. Referrals significant with Bonferroni correction 0.006. MUEPS significant with Bonferroni correction 0.008.

Table 4 highlights the prevalence of various oral symptoms, worst symptoms, symptom patterns, and sites involved. As expected, a burning sensation was present in all BMS patients and absent in non‐BMS patients. Notably, xerostomia was more common in BMS patients (69.9% vs. 50.6%, *p* = 0.017), while oral foreign body sensation was more frequent in non‐BMS patients (26.5% vs. 10.8%, *p* = 0.016). When considering the worst symptoms reported, dysgeusia was more reported in the non‐BMS group (12% vs. 1.2%, *p* = 0.009).

**TABLE 4:** Prevalence of oral symptoms, worst symptoms, localization and symptom pattern in the samples of patients.

	BMS	Non‐BMS	
**Symptoms**	**Frequency (%)**	**Frequency (%)**	***p***
Burning	83 (100)	0 (0)	—
Xerostomia	58 (69.9)	42 (50.6)	0.017
Dysgeusia	36 (43.4)	38 (45.8)	0.876
Globus pharyngeus	34 (41)	29 (34.9)	0.522
Oral dysmorphism	23 (27.7)	23 (27.7)	1.000
Sialorrhea	22 (26.5)	19 (22.9)	0.719
Tingling sensation	9 (10.8)	6 (7.2)	0.590
Oral foreign body sensation	9 (10.8)	22 (26.5)	0.016
Occlusal Dysaesthesia	8 (9.6)	10 (12)	0.804
Oral dyskinesia	7 (8.4)	12 (14.5)	0.330
Hypoesthesia	4 (4.8)	5 (6)	1.000
Subjective Halitosis	3 (3.6)	5 (6)	0.720
Dysosmia	3 (3.6)	7 (8.4)	0.328
Itching	2 (2.4)	5 (6)	0.443
Allodynia	1 (1.2)	3 (3.6)	0.620
**Worst symptoms**	**Frequency (%)**	**Frequency (%)**	
Burning	65 (78.3)	0 (0)	—
Oral dysmorphism	5 (6)	5 (6)	1.000
Xerostomia	4 (4.8)	8 (9.6)	0.370
Sialorrhea	2 (2.4)	2 (2.4)	1.000
Oral dyskinesia	2 (2.4)	1 (1.2)	1.000
Dysosmia	2 (2.4)	2 (2.4)	1.000
Occlusal Dysaesthesia	1 (1.2)	4 (4.8)	0.367
Dysgeusia	1 (1.2)	10 (12)	0.009
Globus pharyngeus	0 (0)	1 (1.2)	1.000
Oral foreign body sensation	0 (0)	4 (4.8)	0.120
Hypoesthesia	0 (0)	1 (1.2)	1.000
**Localization**	**Frequency (%)**	**Frequency (%)**	
Tongue	78 (94)	61 (73.5)	**0.001** ^a^
Lips	61 (73.5)	38 (45.8)	**< 0.001** ^a^
Palate	58 (69.9)	37 (44.6)	**0.002** ^a^
Gums	57 (68.7)	42 (50.6)	0.026
Labial commissure	54 (65.1)	27 (32.5)	**< 0.001** ^a^
Cheeks	54 (65.1)	28 (33.7)	**< 0.001** ^a^
Floor of the Mouth	53 (63.9)	30 (36.1)	**0.001** ^a^
Retromolar trigone	52 (62.7)	25 (30.1)	**< 0.001** ^a^
**Symptom Pattern**	**Frequency (%)**	**Frequency (%)**	
Worse in the evening	39 (47)	40 (48.2)	1.000
Worse in the morning	2 (2.4)	2 (2.4)	1.000
No circadian changes	35 (42.7)	42 (50.6)	0.350
Continuous	45 (54.2)	49 (59)	0.639
Intermittent	33 (39.8)	32 (38.6)	1.000
Worse with meals	30 (36.1)	24 (28.9)	0.408

*Note:* A significant difference between the percentages was measured by Fisher's exact test.

Abbreviations: BMS, burning mouth syndrome; non‐BMS, non burning mouth syndrome.

^a^ Symptoms significant with Bonferroni correction *p* ≤ 0.004. Worst symptoms significant with Bonferroni correction *p* ≤ 0.005. Localization significant with Bonferroni correction *p* ≤ 0.006. Symptom pattern significant with Bonferroni correction *p* ≤ 0.008.

Table S1 presents the prevalence and types of oral symptoms (1, 2, 3 and 4) in patient samples without considering burning. Indeed, burning alone is reported by only 6 patients (7.2%) of the BMS group, suggesting that in BMS, additional oral symptoms are reported in most patients along with burning. When considering additional symptoms, xerostomia was the most associated symptom in the BMS group across all categories (1 symptom, 2 symptoms, 3 symptoms, and 4 symptoms). Dysgeusia was more prevalent among patients with 4 symptoms in the BMS group (86.7% vs. 41.7%, *p* = 0.014). Dysgeusia and xerostomia were common combinations in the BMS group. Considering the number of symptoms present in each patient, it is highlighted that 16 BMS had only one symptom in addition to burning: 12 of them had xerostomia, 2 had dysgeusia, 1 had oral dysmorphic disorder and 1 had globus pharyngeus. In the non‐BMS group, instead, xerostomia and dysgeusia were described, as the only symptom, by 5 and 4 patients respectively while 2 patients had only itching and 1 patient reported only sialorrhea. When two symptoms, excluding burning, were described, globus/xerostomia and dysgeusia/xerostomia were the most common associations in both groups with 32.5% BMS and 22.9% non‐BMS reporting globus/xerostomia and 30% BMS and 24% non‐BMS reporting dysgeusia/xerostomia.

The most prevalent symptom combinations in the BMS group included dysgeusia and xerostomia (25; 30.12%), dysgeusia and globus pharyngeus (17; 20.48%), and dysgeusia and sialorrhea (13; 15.66%). However, none of these combinations reached statistical significance when compared to the non‐BMS group, with *p*‐values of 0.187, 0.500, and 0.228, respectively. Regarding sialorrhea, the combination of sialorrhea and oral foreign body sensation was significantly more common in the non‐BMS group (9; 10.84%) compared to the BMS group (1; 1.2%), with a *p*‐value of 0.007. Other symptom combinations, such as globus pharyngeus and oral foreign body sensation, were also more prevalent in the non‐BMS group (12; 14.46%) compared to the BMS group (4; 4.82%), with a *p*‐value of 0.024. This suggests some but not statistically significant difference in the symptomatology between the two groups. Additionally, combinations involving rarer symptoms like itching, allodynia, and burdensome pain did not show significant prevalence in either group.

Overall, while most symptom combinations did not show significant differences between BMS and non‐BMS groups, specific combinations such as sialorrhea and oral foreign body sensation and globus pharyngeus and oral foreign body sensation were more prevalent in the non‐BMS group, highlighting the importance of detailed symptom assessment in these patients.

## 5 Discussion

This study compares non‐BMS to traditional BMS patients, aiming to better characterise this subgroup. Previously, the 2021 international Delphi study [32] recommended renaming BMS to “Burning Mouth Disorder” to better capture patients' clinical experiences and it suggests that “dysesthesia” could more accurately capture the complex sensations patients report, which are often altered but not always painfully burning, although references to oral burning in the diagnostic criteria remain central. Unlike traditional BMS patients, non‐BMS patients exhibit various dysaesthetic and perceptual symptoms without the hallmark burning sensation of BMS. We acknowledge that classifying this group as non‐BMS may not align with the current diagnostic criteria of ICOP 2020 and ICHD‐3, which require the presence of a burning sensation for a BMS diagnosis [4, 33]. However, the study results indicate that the two groups—non‐BMS and BMS—apart from the absence of burning sensation, are remarkably similar in all examined clinical features. In this study, only 6 patients (7.2%) in the BMS group reported burning as their sole symptom, indicating that additional oral symptoms, including oral dysmorphism, are commonly present and should be carefully evaluated in all patients, regardless of whether they experience burning or not. Both BMS and non‐BMS groups share similar symptom profiles, suggesting the importance of comprehensive symptom assessment in both groups to ensure accurate diagnosis and management.

Both groups showed no significant differences in demographics, habits, or systemic disease prevalence (except for higher rates of hypercholesterolemia and gastroesophageal reflux disease in BMS patients). Both faced diagnostic delays (12–36 months) and consulted similar specialists. Anxiety, depression and sleep disturbances were common in both, though non‐BMS patients reported more daytime sleepiness.

Although non‐BMS patients lack burning sensation, both groups exhibited high NRS and SF‐MPQ scores, indicating reflecting substantial discomfort. The higher SF‐MPQ score in BMS patients suggests the burning sensation intensifies pain perception, making it a more dominant symptom. This highlights the significant impact of the burning sensation on the overall quality of pain perception in BMS patients [34].

Both groups reported similar oral symptoms, with xerostomia, dysgeusia, and globus being the most prevalent. A notable finding is the high prevalence of oral dysmorphism, observed in 23 patients (27.7%) in both groups. These results align with our previous findings [6] who reported this symptom in 108 out of 500 patients with BMS accounting for 21.6% of the sample. This suggests that oral dysmorphism is a common symptom among individuals suffering from BMS. The multifaceted symptomatology associated with the non‐BMS group suggests that this group experiences a narrower and less intense range of sensory disturbances, making it challenging to identify a distinct “worst” symptom, as none reach the severity typically observed in BMS.

Oral dysmorphism refers to perceived abnormal visual changes, in the shape, size, colour or structure of parts of the mouth that are not objectively present upon clinical examination [35]. Patients may report perceived sensations such as an enlarged tongue or gums, changes in the morphology or colour of the gums and tongue, or visual distortions with the most frequent being an enlargement and white discoloration of the tongue, often noticed through frequent self‐examination. This persistent belief in the presence of oral deformities can cause significant distress and impact the person's quality of life. Despite the distress experienced by patients who believe they have a serious condition, the subjective perceptions of structural abnormalities in the mouth are primarily visual and are not necessarily associated with unpleasant sensations; thus, they should not be classified as dysaesthetic symptoms. The phenomenon of visual dysperception is somewhat analogous to Alice in Wonderland Syndrome (AIWS), where patients experience visual distortions, such as changes in size and shape [36]. However, unlike AIWS, where symptoms are primarily visual, non‐BMS and BMS patients exhibit multiple sensory modalities, including salivary (xerostomia and sialorrhea), taste (dysgeusia), tactile (tingling, itching, allodynia), proprioceptive (oral foreign body sensations, occlusal dysaesthesia) and hallucinatory changes (dysosmia and subjective halitosis), making it a more complex condition.

Recently, Farag et al. proposed using only the definition of Oral Dysaesthesia (OD) in current clinical practice instead of BMS [37]. OD is characterised by a persistent alteration in oral sensation, perceived by the patient as abnormal and/or unpleasant, in the absence of identifiable mucosal pathology. They identified BMS as the most encountered idiopathic oral dysaesthesia in a dental setting and they have considered BMS just one of the conditions under the umbrella of OD. However, the authors did not specify all possible oral symptoms associated with burning. Still, they focused only on sensory disturbances beyond pain, such as numbness and tingling, pricking or a shooting sensation. All these symptoms are generally typical of neuropathic pain and unpleasant [38]. It is important to note that sensory disturbances are also observed in nociplastic pain, where they are thought to result from altered central processing rather than direct neural damage, as in neuropathic pain. This highlights the complexity of sensory experiences in OD, potentially involving both nociplastic and neuropathic mechanisms [39, 40].

In this study, along with burning, tingling, hypoesthesia and allodynia, a significant number of additional oral symptoms were reported, like xerostomia, sialorrhea, dysgeusia, globus pharyngeus, oral foreign body sensations, occlusal dysaesthesia, oral dyskinesia, dysosmia and subjective halitosis [9]. Patients frequently report these symptoms, confounding clinicians who cannot make a diagnosis, particularly in the absence of burning [6]. This complex symptom pattern is not generally better characterised in previous studies or in the current classification criteria [41]. Notably, in line with the study of Mignogna et al. [17] in this study also in the BMS group, burning alone is reported by only 6 patients (7.2%), suggesting that additional oral symptoms should be carefully evaluated in all patients with or without burning.

Specifically, while xerostomia and dysgeusia are frequently studied and included in the classification criteria [33] and their etiopathogenesis is directly connected with the neuropathic etiopathogenesis of the disease [42, 43], other symptoms are poorly studied and evaluated [9].

Indeed, oral symptoms such as oral dysmorphism, globus pharyngeus, oral foreign body sensations, occlusal dysaesthesia, oral dyskinesia, dysosmia and subjective halitosis are frequently reported by patients confounding clinicians who cannot make a diagnosis, particularly in the absence of burning.

The oral foreign body sensation refers to the feeling of having a foreign object in the mouth without any physical evidence of such an object being present; sometimes patients may report the presence of coils, wires, insects, or other materials, with no physical evidence. Similarly, globus pharyngeus, also known as globus hystericus, is characterised by the sensation of having a lump or foreign body in the throat without any actual obstruction [44]. These symptoms are defined by several authors as “oral cenestopathy” that include in this definition every abnormal and bizarre sensations in the oral cavity, without any corresponding physical abnormalities [45]. Oral cenestopathy is classified under ‘delusional disorder, somatic type’ or ‘somatoform disorder’ according to the DSM‐5 and is associated with high levels of anxiety and depression [46]. Moreover, recent research indicates that non‐motor symptoms, including cenestopathic sensations, can manifest during the prodromal phase of Parkinson's Disease and precede the classical motor symptoms (like bradykinesia, rigidity, and tremor) by several years [47]. These symptoms, especially when paired with dysosmia, dysgeusia, or oral movement disorders like tongue dyskinesia, require careful evaluation. Dysgeusia and dysosmia are interconnected as taste and smell jointly influence flavour perception. In this sample, 76 reported dysgeusia, 10 dysosmia, and 6 both. Oral foreign body sensation paired with dysgeusia occurred in 20.48% of BMS and 21.69% of non‐BMS patients, while globus and dysgeusia were seen in 7.23% and 10.84%, respectively. Dysgeusia and dyskinesia, or globus and dyskinesia, in either group may indicate serious neurological diseases, emphasising the importance of early recognition for better outcomes.

Occlusal dysaestesia is characterised by the sensation of an uncomfortable or incorrect bite without any physical occlusal discrepancy; it is often associated with emotional distress and can be triggered by dental procedures. Both BMS and occlusal dysaestesia involve sensory alterations where normal sensory signals are misinterpreted as pain or discomfort [48]. This suggests a possible overlap in their pathophysiological mechanisms involving neuropathic pain and maladaptive neural processing.

Subjective halitosis, also known as pseudo‐halitosis is characterised by a perceived foul odour without objective evidence. Similarly to oral dysmorphism this symptom despite highly distressing for the patients is not generally associated with unpleasant sensations, and may be considered as a olfactory hallucination similarly to dysosmia symptom where the individual experiences a sensory perception of smell that does not exist [49, 50]. However, in the study of Bergdahl & Bergdahl [51], the subjective oral dryness and taste disturbances, can contribute to the perception of halitosis, suggesting a neuropathic etiopathogenesis of this symptom. Similarly, also in this study in the majority of cases subjective halitosis was associated with xerostomia and dysgeusia. These changes have an impact on the loss of eating pleasure due to BMS significantly impacts quality of life, aligning with our observations. In BMS patients, pain affects not only sensory perception but also carries a notable emotional and motivational burden.

The study emphasises that the two groups of patients exhibit similar characteristics; thus, it would be beneficial to develop a definition that encompasses both patients experiencing burning sensations and those who do not. It is essential to consider that in our initial patient cohort, 80% reported burning as their primary symptom, and historically, BMS has been the term consistently used to describe this condition [4, 33]. Consequently, altering this well‐established definition may not be appropriate. However, there is a need for a definition that includes patients who do not present with burning but exhibit other oral sensations. Therefore, we have conceptualised the definition of “Oral Dysaesthetic and Perceptual Disorder (ODPD)” for this group of patients who do not fit neatly into the existing diagnostic criteria for BMS. The term ODPD recognises that the broad spectrum of a patient's symptomatology could encompass both dimensions of altered subjective perception (perceptual) and unpleasant physical sensation (dysaesthetic) without corresponding clinical evidence of anomalies.

In Table 5 the definition and descriptions of ODPD are summarised, highlighting both the sensory and perceptual components of the symptoms.

**TABLE 5:** Definition of Oral Dysaesthetic and Perceptual Disorders (ODPD) and symptoms description.

**ODPD definition**	Oral Dysaesthetic and Perceptual Disorder (ODPD) is a chronic condition characterised by persistent sensory and perceptual alterations in the oral cavity. These conditions involve both abnormal sensory experiences (dysaesthesia) and altered perceptions (perceptual changes) affecting the patient's mouth and related structures.
**Dysaesthetic Disorder**	A dysaesthetic disorder is characterised by abnormal and often unpleasant sensations such as burning, tingling, or a feeling of “pins and needles,” occurring without any apparent physical cause whether spontaneous or evoked. These sensations of chronic discomfort can be persistent and debilitating, affecting various parts of the body and significantly impacting daily functioning. This disorder involves sensory disturbances where discomforting sensations are experienced without a clear external stimulus, often described as painful or irritating.
	**Appropriate for**: Symptoms involving abnormal and unpleasant physical sensations typically related to conditions causing sensory dysfunction Examples: pain, burning, allodynia, hypoesthesia, tingling, itching
	**Not appropriate for:** Symptoms that are purely perceptual and do not involve unpleasant sensory components Examples: oral dysmorphism, dysosmia, subjective halitosis
	**Symptoms and Description** **Burning Sensation**: Persistent oral discomfort without visible lesions **Xerostomia**: Dry mouth sensation not necessarily correlated with salivary gland dysfunction **Dysgeusia**: Altered taste perception, such as metallic or bitter tastes **Tingling Sensation**: Abnormal tingling or ‘pins and needles’ in the mouth **Hypoesthesia**: Reduced sensation in the oral cavity. **Dysosmia**: Altered smell perception, such as like a rotten odour or chemicals like ammonia **Itching**: Unpleasant itching sensation in the mouth. **Allodynia**: Pain response to normally non‐painful stimuli
**Perceptual Disorder**	A perceptual disorder is characterised by an impairment in the ability to accurately interpret sensory information, particularly related to immediate sensory experiences, without necessarily involving an abnormal sensory component. This condition results in misinterpretations or distortions of sensory stimuli, including difficulties in recognising objects, sounds, or spatial awareness. These disorders are a type of cognitive impairment that affects the processing of sensory inputs, leading to challenges in understanding or interacting with the environment effectively. Perceptual disorders are characterised by how the brain interprets sensory information disrupting normal sensory perception—such as visual, auditory, or tactile processing—significantly impacting daily functioning. *Examples include hallucinations (perception of non‐existent stimuli), illusions (distortion of real stimuli), and body dysmorphic disorder (distorted perception of one's body).*
	**Appropriate for:** Symptoms involving altered subjective perception without necessarily involving physical pain or discomfort Examples: visual subjective changes (oral dysmorphism), olfactory hallucinations (subjective halitosis, dysosmia), and taste disturbances (dysgeusia)
	**Not appropriate for:** Symptoms involving unpleasant physical sensations such as pain, burning, allodynia, hypoesthesia, tingling, or itching
	**Symptoms and Description** **Globus Pharyngeus**: Sensation of a lump in the throat without physical obstruction **Oral Dysmorphism**: Perceived changes in oral structure without clinical evidence **Sialorrhea**: Excessive salivation perception **Oral Foreign Body Sensation**: Persistent feeling of a foreign object in the mouth **Occlusal Dysaesthesia**: Perception of malocclusion despite normal dental alignment **Oral Dyskinesia**: Involuntary orofacial movements **Subjective Halitosis**: Perceived bad breath without objective confirmation
**Both Dysaesthetic and Perceptual Symptoms**	These symptoms have components of both abnormal sensory experiences (dysaesthetic) and altered perceptions (perceptual). The discomfort or irritation is coupled with a significant perceptual distortion of normal sensory functions.
	**Globus Pharyngeus**: Both the sensation of discomfort (dysaesthetic) and the false perception of a lump (perceptual) **Oral Dysmorphism**: Sensory discomfort (dysaesthetic) and perceived structural changes (perceptual) **Sialorrhea**: Sensory discomfort of excessive saliva (dysaesthetic) and distorted perception of salivation (perceptual) **Oral Foreign Body Sensation**: Sensory irritation (dysaesthetic) and the false perception of a foreign object (perceptual) **Occlusal Dysaesthesia**: Discomfort from perceived malocclusion (dysaesthetic) and perception of malocclusion without physical basis (perceptual)

*Note:* Table references: 1. Merriam‐Webster Medical Dictionary. 2. Mosby's Medical Dictionary. 3.Dorland's Illustrated Medical Dictionary. 4.Stedman's Medical Dictionary. 5.Taber's Cyclopedic Medical Dictionary.

According to medical dictionaries, “perceptual” refers to phenomena involving the perception of sensory stimuli. Perceptual disorders encompass a range of conditions that affect how individuals perceive sensory information. Perceptual alteration can be defined as when there is a change in the pattern of sensory stimuli, followed by an abnormal response to such stimuli. Such perceptions could be increased, decreased or distorted, affecting the patient's hearing, vision, touch sensation, smell or kinesthetic responses to stimuli. Conditions such as oral dysmorphism, subjective halitosis or dysosmia, and dysgeusia fit within this category, where patients perceive changes or sensations that lack objective clinical evidence [52]. “Dysaesthetic” on the other hand, pertains to unpleasant abnormal sensations originating from sensory nerve dysfunction, such as burning, tingling or itching, typically found in neuropathic conditions [53]. This inclusive term effectively covers the range of symptoms experienced by patients, recognising the complexity and multifaceted nature of the patient's symptomatology, and providing a more comprehensive framework for diagnosis and treatment.

The term perceptual is already used frequently in several psychiatric conditions and in other medical contexts. Staab et al. described Persistent Postural‐Perceptual Dizziness (PPPD), a chronic condition characterised by vestibular symptoms like dizziness and instability without identifiable physical causes [54].

The etiopathogenesis of dysaesthetic and perceptual changes is not completely known but could arise from abnormal stress responses. Biochemical imbalances, such as increased levels of dopamine, cortisol, glutamate and norepinephrine, along with reduced cholinergic function, contribute to these changes. Alterations in serotonin or gamma‐aminobutyric acid (GABA) activity also result in various clinical symptoms [55, 56]. Age‐related changes in neurotransmission and cellular signalling can further affect perception [57]. Acute stressors like environmental changes, trauma, severe illness and surgery may disrupt neurotransmitter release, leading to sensory alterations [58]. Additionally, decreased cerebral oxidative metabolism can cause abnormal neurotransmitter release, resulting in brain dysfunction [59]. The cellular signalling hypothesis suggests that disruptions in intraneuronal signal transduction affect neurotransmitter production and release, altering sensation [60].

However, this study has certain limitations that should be acknowledged. Firstly, the study was conducted at a single institution, which may limit the generalizability of the findings to other settings and populations. Additionally, although the study aimed for a sample size sufficient to achieve high statistical power, the final sample of 166 patients (83 in each group) may still be too small to generalise the findings to a broader population, and while the SF‐MPQ was used for sample size estimation as it represents the most appropriate and only tool currently available in the literature to evaluate this aspect comprehensively, we acknowledge that its lack of disease specificity questionnaire a limitation of our study. The study design did not include a longitudinal follow‐up, which restricts the ability to observe changes in symptoms and the effectiveness of treatments over time. Furthermore, the study did not extensively consider cultural and socioeconomic factors that might influence the prevalence and presentation of BMS and non‐BMS, thereby limiting the applicability of the findings across diverse populations. Lastly, as most studies on BMS report a higher prevalence among females, our findings—derived from a more balanced gender distribution achieved through case–control matching—may have limited generalizability to typical clinical populations, where females are often overrepresented in BMS cases.

In conclusion, this study investigated the profiles of patients with non‐BMS compared to traditional BMS patients, aiming to better characterise this subset. Despite the absence of the hallmark burning sensation, non‐BMS patients exhibit a range of dysaesthetic and perceptual symptoms closely aligning with those experienced by traditional BMS patients. The symptomatology of non‐BMS is complex, including dysperceptive and sensory disturbances such as xerostomia, dysgeusia, globus pharyngeus, oral dysmorphism, sialorrhea, tingling sensation, oral foreign body sensation, occlusal dysaesthesia, oral dyskinesia, hypoesthesia, subjective halitosis, dysosmia, itching and allodynia. Therefore, introducing the term “Oral Dysaesthetic and Perceptual Disorder (ODPD)” may provide a more inclusive framework for diagnosing and treating patients who do not fit neatly into existing BMS criteria.

In light of the clinical similarities identified between BMS and non‐BMS patients in this study, we acknowledge that a unified diagnostic category encompassing both burning and non‐burning oral dysaesthetic and perceptual symptoms could offer a more comprehensive approach for future clinical practice. However, due to the limited sample size and current diagnostic conventions, altering the current BMS diagnostic criteria at this stage may be premature. Future updates to classification criteria should include the full spectrum of orofacial dysaesthetic symptoms. Research should focus on understanding the etiopathogenesis of BMS and non‐BMS, developing standardised assessment tools, and conducting larger, longitudinal, multicentric studies to address diagnostic challenges, symptom progression, and treatment outcomes. Current BMS treatments commonly involve antidepressants, antiepileptics, and cognitive behavioural therapy. For non‐BMS patients, similar therapeutic options may be considered, with adjustments based on individual symptoms and specific patient needs. Future studies will help clarify whether a unified treatment approach is effective for both groups or if tailored strategies are required to address their distinct profiles. Developing educational programs and support networks to help patients manage symptoms and learn coping strategies is crucial. Advancing research in these areas will improve understanding, diagnosis, and treatment of BMS and non‐BMS, benefiting patients with these challenging conditions.

## Author Contributions

**Gennaro Musella:** data collection, supervision, manuscript drafting and editing; **Federica Canfora:** study design, data collection, manuscript drafting and editing; **Vito Carlo Alberto Caponio:** data interpretation and manuscript drafting; **Emmanouil Vardas:** data collection and manuscript writing; **Maria Kouri:** data interpretation and data collection; **Nikolaos Nikitakis:** critical revision of the manuscript and data curation; **Giuseppe Troiano:** data interpretation and data validation; **Massimo** **Aria:** data curation, software, data analysis, formal analysis, project administration, manuscript writing; **Luca D'Aniello:** software, formal analysis, data analysis, project administration, data curation, manuscript drafting; **Lorenzo Lo Muzio:** overall supervision of the project and final critical review of the manuscript; **Michele Davide Mignogna:** study conception and final critical review of the manuscript; **Daniela Adamo:** overall supervision of the project, manuscript drafting, final approval of the manuscript, and contribution to the study design.

## Conflicts of Interest

The authors declare no conflicts of interest.

### Peer Review

The peer review history for this article is available at https://www.webofscience.com/api/gateway/wos/peer‐review/10.1111/joor.13945.

## Supporting information


Data S1.

## Data Availability

The datasets generated and analysed during the current study are available from the corresponding author (Federica Canfora), on request. Any material used in the study can be provided upon request in accordance with institutional policies and ethical guidelines.
