# Supplementary material for: Oral Dysaesthetic and Perceptual Disorder, A Distinct Subset of Chronic Orofacial Pain Without Burning Symptoms: A Case–Control Study
Source: J Oral Rehabil. 2025 Jan 27;52(5):651–66. doi: 10.1111/joor.13945 (PMC12037929; doi:10.1111/joor.13945)
Supplement: Supplementary file 1 — Data S1. [file JOOR-52-651-s001.docx]

1. **TABLE S1 | Analysis of the prevalence and type of 1, 2,3 and 4 oral symptoms in the samples of patients**

|  | BMS | non-BMS |  |
| --- | --- | --- | --- |
| Number of patients* | **Frequency (%)** | **Frequency (%)** | **P-value** |
| 1 Symptom | **16 (19.3)** | **12 (14.5)** |  |
| - Xerostomia | 12 (75) | 5 (41.7) | 0.118 |
| - Dysgeusia | 2 (12.5) | 4 (33.3) | 0.332 |
| - Globus pharyngeus | 1 (6.2) | 0 | 0.398 |
| - Oral dysmorphism | 1 (6.2) | 0 | 0.398 |
| - Sialorrhea | 0 | 1 (8.3) | 0.219 |
| - Itching | 0 | 2 (16.7) | 0.076 |
| - Dysosmia | 0 | 0 | - |
| - Oral foreign body sensation | 0 | 0 | - |
| - Allodynia | 0 | 0 | - |
| - Subjective halitosis | 0 | 0 | - |
| - Tingling sensation | 0 | 0 | - |
| - Occlusal dysaesthesia | 0 | 0 | - |
| - Oral dyskinesia | 0 | 0 | - |
| - Hypoesthesia | 0 | 0 | - |
| 2 symptoms* | **23 (27.7)** | **20 (24.1)** |  |
| - Xerostomia | 13 (56.5) | 9 (45) | 0.451 |
| - Globus pharyngeus | 9 (39.1) | 5 (25) | 0.531 |
| - Sialorrhea | 8 (34.8) | 4 (20) | 0.281 |
| - Dysgeusia | 7 (30.4) | 10 (50) | 0.191 |
| - Oral dysmorphism | 3 (13) | 2 (10) | 0.756 |
| - Oral foreign body sensation | 2 (8.7) | 3 (15) | 0.520 |
| - Occlusal dysaesthesia | 1 (4.3) | 1 (5) | 0.919 |
| - Itching | 1 (4.3) | 1 (5) | 0.919 |
| - Dysosmia | 1 (4.3) | 0 | - |
| - Subjective halitosis | 0 | 2 (10) | - |
| - Allodynia | 0 | 1 (5) | - |
| - Oral dyskinesia | 0 | 1 (5) | - |
| - Tingling sensation | 0 | 0 | - |
| - Hypoesthesia | 0 | 0 | - |
| 3 symptoms* | **14 (16.8)** | **16 (19.8)** |  |
| - Xerostomia | 12 (85.7) | 12 (75) | 0.451 |
| - Dysgeusia | 9 (64.8) | 8 (50) | 0.191 |
| - Globus pharyngeus | 8 (57.1) | 6 (37.5) | 0.531 |
| - Oral dysmorphism | 6 (42.9) | 4 (25) | 0.756 |
| - Sialorrhea | 4 (28.7) | 5 (31.2) | 0.281 |
| - Dysosmia | 1 (7.1) | 1 (6.2) | 0.345 |
| - Occlusal dysaesthesia | 1 (7.1) | 3 (18.7) | 0.919 |
| - Oral dyskinesia | 1 (7.1) | 3 (18.7) | 0.278 |
| - Oral foreign body sensation | 0 | 4 (25) | - |
| - Allodynia | 0 | 1 (6.2) | - |
| - Itching | 0 | 1 (6.2) | - |
| - Subjective halitosis | 0 | 0 | - |
| - Tingling sensation | 0 | 0 | - |
| - Hypoesthesia | 0 | 0 | - |
| 4 Symptoms* | **15 (18) (** | **12 (14.5)** |  |
| - Xerostomia | 13 (86.7) | 7 (58.3) | 0.095 |
| - Dysgeusia | 13 (86.7) | 5 (41.7) | 0.014 |
| - Globus pharyngeus | 8 (53.3) | 8 (66.7) | 0.484 |
| - Oral dysmorphism | 7 (47.7) | 8 (66.7) | 0.299 |
| - Sialorrhea | 4 (26.7) | 5 (41.7) | 0.411 |
| - Occlusal dysaesthesia | 3 (20) | 2 (16.7) | 0.825 |
| - Oral dyskinesia | 3 (20) | 2 (16.7) | 0.825 |
| - Dysosmia | 1 (6.7) | 2 (16.7) | 0.411 |
| - Oral foreign body sensation | 6 (40) | 7 (58.3) | 0.343 |
| - Allodynia | 0 | 1 (8.3) | 0.255 |
| - Subjective halitosis | 2 (20) | 1 (8.3) | 0.681 |
| - Itching | 0 | 0 | - |
| - Tingling sensation | 0 | 0 | - |
| - Hypoesthesia | 0 | 0 | - |

*A significant difference between the percentages was measured by Fisher’s exact test.*

* Without including the burning symptom

***1 Symptom significant with Bonferroni correction 0.008*

***2 Symptoms significant with Bonferroni correction 0.006*

***3 Symptoms significant with Bonferroni correction 0.006*

***4 Symptoms significant with Bonferroni correction 0.005*

***Abbreviation****:* BMS, burning-Burning Mouth Syndrome; non-BMS, non burning-Burning Mouth Syndrome.

**2.** **Symptoms assessment**

- 1. Salivary evaluation

For the assessment of whole salivary flow, experts measured and collected the unstimulated and stimulated salivary flow rates of all patients, following standardized procedures from previous validated studies^1,2^. Flow collection was performed using two separate graduated cylinders: for measuring unstimulated saliva flow, patients were asked to expectorate for 5 minutes, at least one hour after eating, drinking, or chewing gum, while resting in a quiet room; for stimulated saliva flow, patients were instructed to chew on a tasteless paraffin pellet (manufactured by Aurosan GmbH) to promote salivation, and were asked to expectorate for 5 minutes while chewing on the pellet, without swallowing. All salivary collections occurred during the daytime clinical hours between 9:30 am and 4:30 pm. The cut-offs for unstimulated and stimulated saliva flow were set at ≤0.1 mL/min and ≤0.5 mL/min, respectively, and for rates lower, a diagnosis of hyposalivation was assessed ^2,3^.

Ancillary examinations were also performed, including ultrasound imaging of the major salivary glands using high-frequency (7–15 MHz) probes to assess structural integrity and help rule out conditions like acute inflammation or sialolithiasis^4^.

1.2 Allodynia

A slight vibration of a cotton swab was applied to the alveolar mucosa for 10 seconds, and the pain intensity was recorded using a NRS to ensure precision in measurement. The area stimulated by the cotton swab was approximately 2 mm², ensuring a consistent and focused application of the stimulus. This method was chosen to provide a standardized approach for assessing the pain response in the specific area of the alveolar mucosa^5^.

1.3 Hypoesthesia

A mechanical pain threshold test using needle stimulators (pinprick, MRC Systems GmbH, Germany) was assessed to determine hypoesthesia. The needle stimulators employed consist of blunt needles with a fixed stimulation intensity of 8, 16, 32, 64, 128, 256, and 512 mN. These needles have a beveled, circular contact area with a diameter of 0.25 mm. The procedure involves applying the individual needle stimulation devices perpendicularly to the area in five test series, alternating between ascending and descending stimulus intensities. Each application maintains a contact time of approximately 1–2 seconds. This method allows us to calculate the mechanical pain threshold using the “level” method, which determines the geometric mean of the five stimulus intensities just above and five just below the threshold.^6^

1.4 Dysgeusia

To assess dysgeusia, a combination of taste strips and electrogustometry is commonly employed. Taste strips are impregnated with specific tastants—such as sweet, sour, salty, and bitter—and are applied to various regions of the tongue to evaluate the patient's ability to identify different taste qualities. Electrogustometry involves applying a mild electrical current to specific areas of the tongue to directly stimulate taste perception, allowing for the measurement of taste thresholds. This dual approach provides a comprehensive evaluation of taste function, facilitating the detection and quantification of dysgeusia. ^7^

References:

1. Navazesh M, Kumar SK, University of Southern California School of D. Measuring salivary flow: challenges and opportunities. *J Am Dent Assoc.* 2008;139 Suppl:35S-40S.

2. Sreebny LM. Saliva in health and disease: an appraisal and update. *Int Dent J.* 2000;50(3):140-161.

3. Lee YC, Hong IK, Na SY, Eun YG. Evaluation of salivary function in patients with burning mouth syndrome. *Oral Dis.* 2015;21(3):308-313.

4. Aringhieri G, Izzetti R, Vitali S, et al. Ultra-high frequency ultrasound (UHFUS) applications in Sjogren syndrome: narrative review and current concepts. *Gland Surg.* 2020;9(6):2248-2259.

5. Svensson P, Baad-Hansen L, Pigg M, et al. Guidelines and recommendations for assessment of somatosensory function in oro-facial pain conditions--a taskforce report. *J Oral Rehabil.* 2011;38(5):366-394.

6. Mucke M, Cuhls H, Radbruch L, et al. Quantitative sensory testing (QST). English version. *Schmerz.* 2021;35(Suppl 3):153-160.

7. Zhu Y, Hummel T. Assessment of Taste Function. *Handb Exp Pharmacol.* 2022;275:295-319.
